# The role of cognitive effort in subjective reward devaluation and risky decision-making

**DOI:** 10.1038/srep16880

**Published:** 2015-11-20

**Authors:** Matthew A J Apps, Laura L Grima, Sanjay Manohar, Masud Husain

**Affiliations:** 1 Nuffield Department of Clinical Neuroscience, University of Oxford, Oxford, UK; 2 Department of Experimental Psychology, University of Oxford, Oxford, UK

## Abstract

Motivation is underpinned by cost-benefit valuations where costs—such as physical effort or outcome risk—are subjectively weighed against available rewards. However, in many environments risks pertain not to the variance of outcomes, but to variance in the possible levels of effort required to obtain rewards (effort risks). Moreover, motivation is often guided by the extent to which cognitive—not physical—effort devalues rewards (effort discounting). Yet, very little is known about the mechanisms that underpin the influence of cognitive effort risks or discounting on motivation. We used two cost-benefit decision-making tasks to probe subjective sensitivity to cognitive effort (number of shifts of spatial attention) and to effort risks. Our results show that shifts of spatial attention when monitoring rapidly presented visual stimuli are perceived as effortful and devalue rewards. Additionally, most people are risk-averse, preferring safe, known amounts of effort over risky offers. However, there was no correlation between their effort and risk sensitivity. We show for the first time that people are averse to variance in the possible amount of cognitive effort to be exerted. These results suggest that cognitive effort sensitivity and risk sensitivity are underpinned by distinct psychological and neurobiological mechanisms.

Theoretical perspectives on motivation argue that people obey a “law of least effort”^1^, minimizing the amount of effort they exert in order to obtain desirable outcomes. Past research has shown that many species obey this law in cost-benefit decision-making tasks, with the rewards (benefit) on offer devalued (discounted) by the effort (cost) required to obtain them^2^,^3^,^4^,^5^,^6^. An imbalance in how such cost-benefit evaluations are made has been suggested as a potential mechanism for a number of disorders of motivation, such as apathy and impulsivity^2^,^7^,^8^,^9^ and also the motivational deficits suffered by individuals with neurological conditions, such as Schizophrenia, Parkinson’s and Alzheimer’s disease^10^,^11^. However, motivation also varies considerably in the healthy population^2^,^12^,^13^,^14^. Elucidating the mechanisms that underpin cost-benefit decision-making may therefore be crucial for understanding disorders of motivation and variation in motivation in the healthy population.

Until recently, research examining effort-discounting has overwhelmingly focused on how physical effort devalues rewards. However, emerging evidence suggests that rewards are also devalued when the effort is cognitive, rather than physical. Rats have been shown to forego large rewards in order to avoid cognitive demands in a visuospatial attention task^15^,^16^. Similarly, in humans rewards are subjectively devalued both by the cognitive costs incurred when switching between different tasks and also by the demands of working memory paradigms^17^,^18^,^19^. These valuations are highly idiosyncratic, with some individuals requiring very large rewards to exert effort, but others choosing to exert the same amount of effort for small rewards. However, very little is known about how cognitive effort discounting relates to other forms of cost-benefit evaluation, such as risky decision-making, nor whether it is related to apathy (a reduction in goal-directed behavior), as has been shown for physical effort^2^,^11^.

Risks are a ubiquitous feature of most environments, with very few of our choices having certain outcomes^20^,^21^,^22^,^23^,^24^. Much like effort, risks are also *subjectively* weighed against rewards. Some individuals are risk-averse, choosing safe options with a certain outcome over options where the average amount of reward on offer is equal, but the final outcome could be better or worse than the safe option. In contrast, other individuals are risk-seeking, preferring to gamble on the risky option^25^. A substantial body of research has investigated how rewards are subjectively valued when the risk pertains to the probability of receiving particular magnitudes of rewarding outcomes^20^,^26^. However, in many environments the rewarding outcomes themselves are not risky, but there is variance in the amount of effort that might have to be exerted in order to obtain them^27^. That is, we often have to evaluate whether it is worth putting in a known, moderate amount of effort, or choose a risky option where the amount of effort that might have to be exerted could be very high or very low.

To date only one study has examined how risky-decisions are made when the risk pertains to the amount of effort that may have to be exerted^28^. In Nagengast *et al.*, (2011) choices were made between a fixed amount of physical effort and a risky effort option, where there was variance associated with the effort on offer. Overall people were risk-seeking, and willing to “gamble” on a risky effort option, in order that they might avoid having to exert the moderate amount of effort in the safe option. Whether people are similarly sensitive to risky cognitive effort is unclear. Recent studies have suggested that cognitive effort has a different anatomical and pharmacological signature from physical effort^15^,^16^. However, no previous study has directly examined whether people are averse or sensitive to cognitive effort risks, nor whether it is related to trait measures of risk proneness^29^.

Recently it has been proposed that the neural mechanisms that underpin effort-discounting overlap with those of risky decision-making^30^. Such a common neural currency for effort discounting and risk-sensitivity could lead to a relationship between the two. Specifically, the extent to which an individual subjectively devalues rewards by cognitive effort would be correlated with how sensitive they are to risky cognitive effort. However, the extent to which rewards are devalued by effort has not previously been related to the extent to which individuals are risk-seeking.

In this study, we examined how sensitive participants were to cognitive effort and risky effort in two cost-benefit decision tasks. Cognitive effort was manipulated in a rapid serial visual presentation paradigm (Fig. 1a)^31^, by controlling how many peripheral shifts of attention would be required (between 1–6 shifts). Following extensive training at each level of cognitive effort, participants indicated their preferences on two decision-making tasks that indexed the extent to which rewards were discounted by peripheral shifts of attention (Fig. 1b) and the extent to which variance in the number of shifts of attention would be performed led to risk-aversion or risk-sensitivity (Fig. 1c). We used this devaluation to establish if people are risk-seeking with cognitive effort; and to determine if there is a relationship between the extent to which people idiosyncratically devalue rewards and how sensitive they are to cognitive effort risks.

## Results

**Figure 1:**
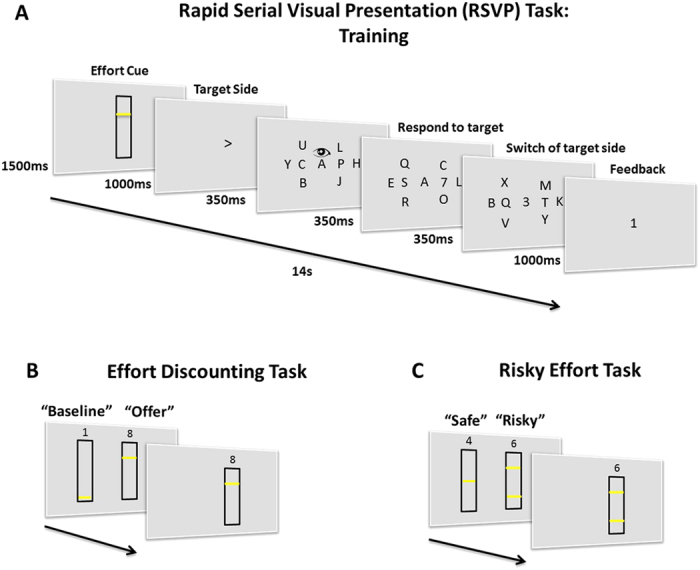
RSVP Trial Structure. (**A**) To manipulate cognitive effort we controlled the number of peripheral shifts of attention in an RSVP task. Participants were required to maintain central fixation as an array of letters changed rapidly and attend to a “target” stream presented horizontally to the left or right of a central stream, in order to detect targets (the number “7”). The initial target side was indicated at the beginning of the trial by an arrow. During each trial a cue in the centre of the screen (a number “3) indicated that the target side was switching, requiring participants to make a peripheral shift of attention. Effort was manipulated by controlling the number of presentations of shift cues from one to six. In the training session feedback was provided in the form of credits (1 credit or 0) at the end of each trial if participants successfully detected a sufficient number of targets. (**B**) **Effort discounting task (EDT)**. Choices were made between a fixed “baseline” and a variable “offer”. The baseline was fixed at the lowest effort and reward (1 credit, 1 shift). The offer varied in terms of reward and effort (2, 4, 6, 8, 10 credits and 2, 3, 4, 5, 6 shifts). Choices on this task indexed the extent to which rewards were devalued by shifts of attention. (**C**) **Risky Effort Task (RET)**. Choices were made between a safe option, with fixed effort and reward levels (3 shifts, 4 credits) and a risky option which varied over trials in terms of reward (2, 4, 6 credits) and risk (low or high). The risky option was associated with a 50% probability of having to perform one of two effort levels that were low (2 or 4 shifts) or high (1 or 5 shifts) in variance. The extent to which the safe option was chosen indexed cognitive effort risk-aversion or risk-sensitivity.

Participants (n = 40) indicated their preferences on two cost-benefit tasks, where the costs related to the amount of amount of cognitive effort required, and the benefit was monetary reward. They were first trained on a rapid serial visual presentation (RSVP) task where they had to fixate centrally but monitor a peripheral stream, either to the left or right of fixation, in order to detect a target (number 7) (Fig. 1a). The central stream of stimuli also had to be monitored for a cue (number 3), which indicated that participants should shift attention, and monitor the contralateral peripheral stream. During a prior learning phase, participants experienced how much effort was required to make 1, 2, 3, 4, 5 or 6 shifts of spatial attention during 14 s trials (see Fig. 1a and Methods). Following this – in a counterbalanced order across participants—an effort-discounting paradigm (EDT) and risky effort task (RET) were performed, where cost-benefit choices were made on the basis of whether a reward was “worth it” (Fig. 1b,c).

### Effort Discounting Task

#### Rewards are devalued by effortful shifts of attention

The effort-discounting task (EDT) allowed us to examine the extent to which rewards are discounted by the number of shifts of spatial attention. In the EDT, participants made choices between a *fixed* low effort, low reward “baseline” (one shift, one credit) and a “higher effort” option that varied in the amount of effort (from 2–6 shifts) and reward (2, 4, 6, 8 or 10 credits) on offer over trials. If the baseline was chosen it indicated that the reward in the offer had been devalued to such an extent it was worth less than the baseline. Thus, participants’ choices on the EDT indexed how much influence the effort was having on their valuation of reward. This study is the first to use shifts of attention to manipulate cognitive effort. It was therefore important to demonstrate firstly that rewards are devalued by the number of shifts of attention and secondly, that a greater number of shifts of attention were perceived as more mentally demanding or subjectively effortful.

**Figure 2:**
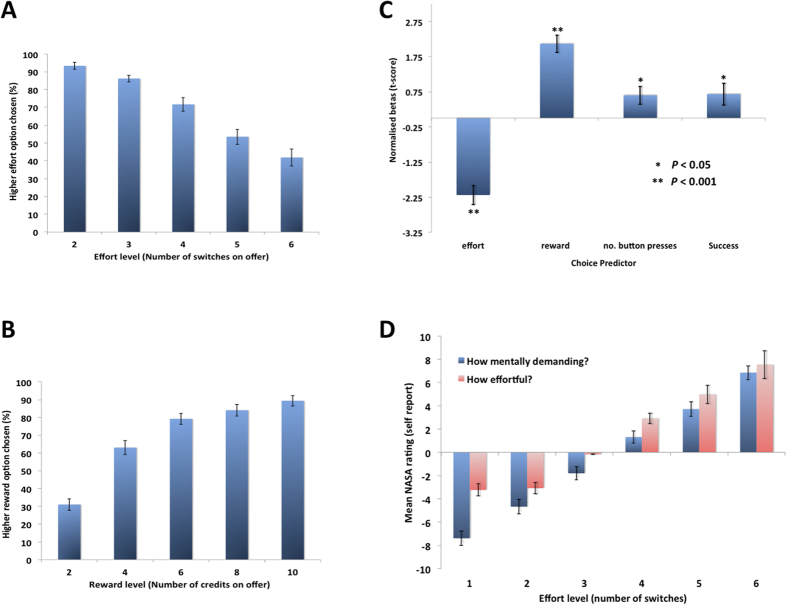
Shifts of attention are effortful and devalue rewards. (**A**) Proportion of trials where the higher effort option (y-axis) was preferred, as a function of the number of shifts of attention in the offer (x-axis). As the number of shifts of attention offered increased, the less likely it was that the offer was chosen. (**B**) Proportion of trials the higher reward option (y-axis) was selected, as a function of the reward on offer (x-axis). As the amount of reward offered increased, the less likely it was that the offer was chosen. (**C**) Results of a logistic regression. Mean normalised betas for predictors of choosing the higher effort, higher reward offer. Effort was a significantly better predictor of choice than two other control predictors, the number of button presses and a task success predictor. (**D**) Results from the NASA-TLX. Participants completed the self-report NASA-TLX for each effort level, rating how demanding they found each number of shifts (x-axis) from −10 to + 10 (y-axis). Crucially, the higher the number of shifts of attention, the more mentally demanding (blue) and effortful (red) the ratings. Error bars depict SEM.

Repeated measures linear ANOVAs showed that both effort and reward significantly influenced choices (main effect of effort: F(1.61,22.55) = 25.590 *p* < .001; main effect of reward: F(2.32, 32.47) = 28.43, *p* < .001). As predicted, as the number of shifts increased in the higher effort option (Fig. 2a,b), the proportion of choices of this “offer” decreased (See supplementary results for further information). These findings are therefore consistent with the notion that shifts of attention in this paradigm are considered to be costly and devalue rewards.

Are shifts of attention subjectively considered effortful? To examine whether shifts of attention are perceived as more mentally demanding and effortful we used the NASA Task Load Index (NASA-TLX)^32^. This self-report measure requires people to rate between “very low” and “very high” on six categories (mental demand, physical demand, temporal demand, effort, frustration and performance). Participants rated each of the six effort levels on each of these categories. Repeated measures linear ANOVAs highlighted a significant effect of effort level on all six categories (*p* < 0.001). Importantly, there were highly significant effects on mental demand and effort (Fig. 2d) indicating that a higher number of shifts of attention as more effortful (Main effect of mental demand: F(1.5,58.7) = 144.2, *p* < 0.001; main effect of effort: F(2.7,102.6) = 101.386, *p* < 0.001). Thus, as the number of shifts of attention increased the perceived amount of effort increased.

#### Reward devaluation is driven by cognitive effort

Reward devaluation can occur due to factors other than cognitive effort such as the amount of physical effort required or errors/poorer task performance^17^. To examine whether decision-making on the EDT was driven by the effects of cognitive effort or other confounding effects we performed a logistic regression on choices in the EDT, with four factors predicting choice, for each participant. The first two predictors were the effort level and the reward level in the offer. The third predictor was the number of button presses made by a participant for the effort level on offer–calculated as the average number of button presses performed for a given effort level in the training session. The fourth predictor was the level of success for the effort level on offer–calculated as the average number of misses and false alarms in the training session at a given effort level, for that participant. These last two predictors therefore allowed us to examine whether physical effort and performance respectively could account for participants’ choices during the experiment.

The resulting beta weights for each predictor were converted into t-scores (beta divided by standard error in the estimate of the beta). The t-scores for the predictors were not normally distributed (Kolmogorov- Smirnov: all *p *< 0.05) and thus Mann-Whitney U tests were performed to examine whether each predictor could significantly explain choice behavior. All four predictors (see Fig. 2c) significantly influenced choices (Effort: U = −5.15, *p* < 0.001; Reward: U = 5.51, *p* < 0.001; Success: U = 2.70, *p* < 0.01; Button presses: U = 2.06, *p* < 0.05). However, Mann-Whitney U tests between predictors showed that effort predicted choice behavior significantly better than the success level or the button presses (Success: U = 2.89, *p* < 0.005; Button Presses: U = 2.55, *p* = 0.01), highlighting that choices were primarily guided by the number of shifts of attention in the offer and thus the amount of cognitive effort required. In an additional analysis using d’scores (hits minus false alarms) rather than the success rate, showed very similar effects. There was also no order effects of whether a participant performed this task before or after the RET. (supplementary results).

### Risky Effort Task

#### Risk-aversion for cognitive effort

The RET required participants to make choices between a fixed, safe baseline (3 shifts, for 4 credits) and a risky option in which the mean amount of effort was the same but the variance was varied across trials. There were three levels of reward in the risky offer, which could be lower, equal or higher than the reward in the safe offer (2,4 or 6 credits). The risky offer was always associated with two effort levels, one which was less effortful than the safe offer, and one which was more effortful, but with the same mean (3 shifts). If the offer was chosen, participants had to perform one of these two effort levels with equal probability. The risky offer was either low in variance (low risk – 2 or 4 shifts) or high in variance (high risk, 1 or 5 shifts). Using this design we were able to examine the effects of reward and variance in cognitive effort on preferences.

**Figure 3:**
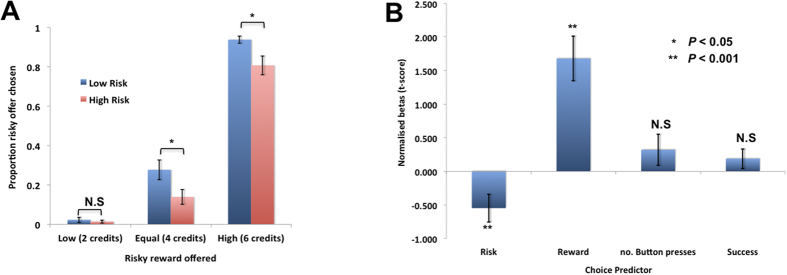
Aversion to cognitive effort risk. (**A**) Proportion of trials on which the risky offer was chosen (y-axis), as a function of the reward level in the risky offer, which was either lower, equal to, or higher than the reward for the safe option (4 credits). We found that people were averse to the risky offers, choosing it on less than 50% of trials even when the reward in the risky offer was equal to the reward in the safe option. Moreover, we showed a risk level x reward interaction highlighting that people were more likely to choose the safe option when the variance in the risky option was high. (**B**) **Logistic regression.** Mean, normalised betas (y-axis) for predictors of choosing the risky effort option, showing an effect of risk level and reward, but no effect of physical effort (mean no. of button presses in training session) and failure rate (a mean of the number of false alarms and misses in the training session). N.S = not significant. Error bars depict SEM.

A 2 × 3 repeated measures ANOVA of Risk (low, high) and Reward (low, equal, high) on the proportion of choices of the risky offer revealed a significant interaction between risk and reward, as well as main effects of both risk and reward (Risk × Reward: F(1.6, 62.4) = 4.76, *p* < 0.05; Risk: F(1.0, 62.4) = 16.33, *p* < 0.001; Reward: F(2.0, 62.4) = 270.29, *p* < 0.001). Post-hoc *t*-tests showed that this effect (Fig. 3a) was driven by significant differences between high and low risk when the reward in the risky offer was high or equal to the baseline, but no effect at the low reward level (High: *t*(39) = 3.074, *p* < 0.01; Equal: *t*(39) = 3.31, *p* < 0.01; Low: *t*(39) = 0.589, *p* > 0.5). In addition, there was no significant interaction between reward and risk when examining only the equal and higher reward levels in a 2 × 2 ANOVA (F(1,39) = 0.016, *p* < 0.9). This suggests that the highest levels of reward do not modulate the effect of effort risk.

These effects were driven by a decreased willingness to choose the risky option when the risks were high, suggesting that people are averse to cognitive effort risks (Fig. 3a). This was further emphasized by the fact that participants were less likely than chance (50%) to choose the risky offer even when the reward offered was equal to the reward in the safe option (Low risk—equal reward: *t*(39) = −4.49, *p* < 0.001; High risk – equal reward: *t*(39) = −9.45, *p* < 0.001). Thus, overall, people were risk averse, preferring to choose the safe option more often than the risky option, and this effect was magnified when there was a variance to the risky offer.

#### Risk-aversion is driven by cognitive effort risks

Can choices on the RET be driven by differences in the expected *physical* – rather than cognitive – effort or success for the two levels of effort in the high-risk option compared to the low risk option? To examine this we used the reward and risk levels on each trial as predictors of choice in logistic regressions, as well as two predictors calculated based on performance in the training session. The two predictors based on performance were the combined average number of button presses, and the combined average number of misses and false alarms, across the two effort levels in the risky offer (2 and 4 shifts or 1 and 5 shifts). This enabled us to examine the effects of physical effort and success on choices respectively. The resulting beta weights for each decision-variable were converted into t-scores (beta divided by the standard error of the estimate of the beta). The t-scores for all the predictors were not normally distributed (Kolmogorov- Smirnov: *p *<* 0.05)* and thus Mann-Whitney U tests were performed to examine whether each decision-variable could significantly explain choice behavior. Reward significantly, positively predicted choice of the risky option, and risk (entered as a dummy variable) significantly, negatively predicted choices of the risky option. As the risk increased there was a greater probability of the safe option being chosen (Risk: U(39) = −3.91, *p* < 0.001; Reward: U(39) = 5.51, *p* < 0.001; Button Presses: U(39) = 0.99, *p* > 0.3; Success: U(39) = −1.15, *p* > 0.2). In addition, the variance in button presses and success also did not significantly predict choice (supp. results). Thus, risky cognitive effort decision-making was driven mainly by the reward on offer and the amount of variance in the cognitive effort offered.

#### No relationship between cognitive effort discounting and risk-aversion

To examine whether the extent to which an individual devalued rewards by cognitive effort was related to the extent to which they were averse to cognitive effort risks, we performed correlations between the t-score from the regressions against choice from the EDT and the RET. We found no significant correlation between the t-scores from the effect of *effort* on decision-making from the EDT and the risk effects from the RET (Spearman’s R_s_ = 0.136, *p* > 0.4). Importantly, this also suggests that choices on the risk experiment – and particularly the aversion to risk—were driven by the variance in the risk and not down to increased sensitivity to the higher effort level present in the high risk offer. However, there was a marginally significant positive correlation between the effects of *reward* in the EDT and the RET (R_s_ = 0.331, *p* = 0.051, two-tailed). This suggests that reward sensitivity across the two tasks is relatively stable, but effort-discounting and risk effects are potentially dissociable from each other. In addition there was no difference in choices on the RET if a participant performed this task before or after the EDT (supplementary results).

#### No relationship between EDT or RET and self-report measures of apathy and risk

Previous studies have shown that discounting on *physical* effort tasks is correlated with behavioural apathy^2^,^33^. To examine whether there was any relationship between effort discounting and self-report levels of apathy in this experiment, we correlated the reward and effort t-scores from the logistic regression performed on choice data on the EDT and scores on the LARS-e (Lille Apathy Ratings Scale extended)^2^,^34^ assessment of apathy. We found no significant correlation between the t-scores from the effect of effort on decision-making from the EDT and the risk effects from the RET (Spearman’s Rs = 0.136, p > 0.4). Importantly, this also suggests that choices on the risk experiment—and particularly the aversion to risk—were driven by the variance in the risk and not down to increased sensitivity to the higher effort level present in the high risk offer. However, there was a marginally significant positive correlation between the effects of reward in the EDT and the RET (Rs = 0.331, p = 0.051, two-tailed). This suggests that reward sensitivity across the two tasks is relatively stable, but effort-discounting and risk effects are potentially dissociable from each other.

#### Cognitive effort is not related to eye movements

**Figure 4:**
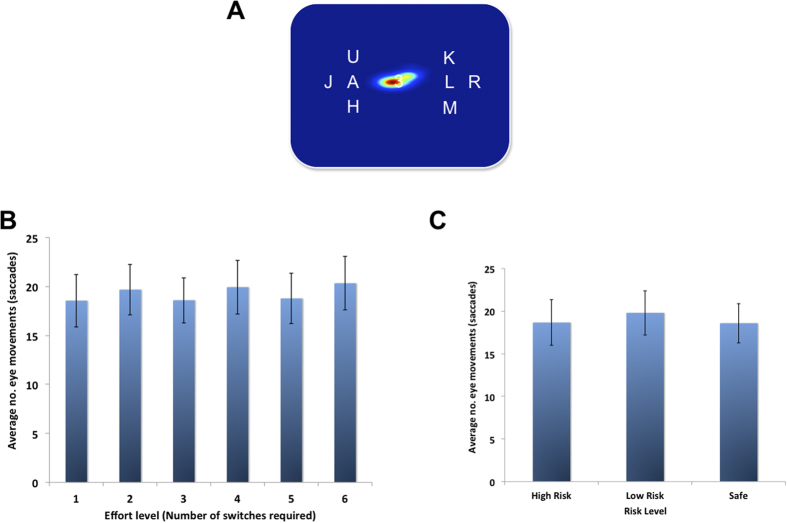
Fixation and saccades during the training session. (**A**) Heatmap showing a histogram of the fixation locations during the training session, overlaid on an array from the RSVP trials. The heat map shows that participants were performing the task as instructed, fixating centrally and not on the target streams. (**B**) Graph of the average number of saccades (y-axis) per each effort level (x-axis), showing that as the number of shifts of attention increased, the mean number of saccades did not. (**C**) Graph of the same data as in (B) but plotted as a function of the effort levels in the safe option (3 shifts), the low risk (2 and 4 shifts) or high risk (1 and 5 shifts). Error bars depict SEM.

Is it possible that when participants were making peripheral shifts of attention, they were also making an increasing number of eye movements across the different cognitive effort levels? To examine this question we performed an additional experiment (n = 14). The same series of training trials were completed as during the main experiment, but the number of saccades and fixation positions were recorded during the 14 s RSVP trials. Using a linear ANOVA we showed no difference in the mean number of saccades across the effort levels (F (2.88, 37.48) = 3.78, *p* > 0.05). In addition, using a linear ANOVA we also showed no difference in the mean number of saccades for the average of the two effort levels in the risky offers (F (1.284, 16.70) = 0.016, *p* > 0.9). Finally, we plotted a histogram of fixation locations as a heatmap (Fig. 4a). This highlights that the vast majority of fixations were central and not at the location of the two peripheral target streams. These results indicate that participants were performing the task as instructed, and also that the varying number of shifts of attention during the RSVP trials does not relate to a varying amount of physical effort that might occur as a function of the number of eye movements made.

## Discussion

We examined whether spatial shifts of peripheral attention are experienced as effortful, devalue rewards and whether people are sensitive to risks in terms of the amount of effort they have to exert. The findings reveal that shifts of attention are experienced as effortful and devalue rewards. Moreover, people ‘play safe’ with cognitive effort risks and do not gamble on risky options where they may end up having to exert either a very high amount or very low amount of cognitive effort. This finding contrasts with previous studies examining physical effort that have reported risk-sensitivity rather than risk-aversion^28^. Thus, we suggest that our results support recent work which proposes that cognitive and physical effort may be underpinned by distinct cognitive and neural mechanisms^15^,^16^.

The notion that cognitive processes are costly and are avoided unless associated with sufficient benefits is an oft-cited principle in psychological research. However, only a handful of studies have systematically explored how rewards are subjectively weighed against cognitive costs^35^. Previous investigations have used a variety of different cognitive tasks to manipulate cognitive effort, including the N-back, task-switching or response inhibition^17^,^18^,^19^. These studies have shown that cognitive effort devalues rewards over and above other factors that can discount rewards^36^, including the amount of physical effort^2^, delay until rewards are received^37^,^38^,^39^,^40^, or avoidance of errors on the task^41^. In our investigation, the length of time during which the effort was exerted was equal across all effort levels, thus ensuring that the effects reported here could not be driven by rewards being temporally discounted. We also showed that the number of shifts of attention better explained choices than the amount of *physical* effort that would be expected in terms of button presses, or the numbers of errors they were expecting. Thus shifts of spatial attention to monitor a rapid stream of visual presentation are considered by people to be effortful and devalue rewards over and above other factors that discount rewards.

Whilst some studies have examined the devaluation of rewards by cognitive effort, no previous investigation has ruled out the possibility that the reward devaluation effects observed may have been driven by an alternative source of physical effort: eye movements. Past research has reported that saccades are costly actions that devalue rewards^42^,^43^. Thus, for the devaluation of rewards to be wholly driven by cognitive effort, the number of eye movements across the different effort levels would need to be consistent. Here, we were able to show that as the number of shifts of peripheral attention increased, the number of saccades did not, consistent with the view that effort-discounting effects may be driven by the costs of cognitive processes engaged and not alternative physical costs of saccadic movements.

What is costly about peripheral shifts of attention? We suggest three possible causes for why shifts of attention devalue rewards. The first is that such shifts to alternative spatial locations may come at a time cost that devalues the reward, as it would be detrimental towards performance^43^. In our task, such time costs would increase the probability of missing target stimuli and therefore potentially decrease the likelihood of being rewarded. Whilst this is a possible explanation, we found the amount of effort and risk in the two decision-making experiments significantly accounted for choices better than actual measures of performance. This would not be in keeping with a temporal devaluation account in which it would be predicted that performance would be significantly worse. Moreover, we used an adaptive approach such that rewards were delivered approximately equally at each level of effort. As such, it is unlikely that the significant difference in valuation between different effort levels is driven simply by the time cost of attention shifts.

A second explanation is that it may be effortful to inhibit eye movements. Past research has shown that any stimuli presented peripherally, or cues that explicitly direct attention to alternative spatial locations, automatically afford motor plans for saccades to their locations. The suppression of such motor plans is thought to come at a computational cost^44^,^45^,^46^,^47^. In our task, participants were required to suppress any eye movements and maintain central fixation, whilst centrally presented “shift” cues instructed that they shift their spatial location. Whenever such a cue was presented, the saccadic motor program would have to be inhibited to prevent a saccade to the location of the target stream. Whilst this is a plausible mechanistic account of a potential cost in the RSVP task, we did not find an increase in the number of eye movements as the number of shifts of attention increased. Such an effect would be expected if the centrally presented shift cues were automatically triggering motor plans. Thus, it would seem unlikely that the costs of inhibiting automatically cued motor plans would be sufficient to drive the strong effects we observed on choices in the RET and the EDT.

An alternative explanation is that switching spatial attention, like other shifts in cognitive processes, comes at a significant cost. Previous research that examined cognitive effort has shown that switching between different tasks is subjectively effortful and devalues rewards^17^,^19^,^36^. Moreover, there is evidence of a domain-general brain mechanism underpinning shifts of attention, switching between rules or switching between different stimulus-response mappings^48^. Presumably, shifts of attention require breaking one established task set and reconfiguring to an alternative. One parsimonious account of our effort discounting effect is that shifting attention to a different location comes at a cost in a similar manner to switching between tasks or rules. Switching between different cognitive processes may therefore be highly costly in terms of the neural resources required to make large shifts in the employed task set. As a result, shifts of attention are effortful and avoided unless associated with significant reward. Future research might profitably aim to characterize what exactly is effortful about large-scale switches of task-sets or patterns of behaviour.

This study is the first to show that people are averse to risky cognitive effort, preferring to choose a safe option if the alternative contains a risk of having to exert a high amount of cognitive effort. Importantly this effect is unlikely to be related simply to how aversive the higher levels of effort were, as there was no relationship to how sensitive individuals were to cognitive effort (on the EDT) and how risk-averse they were (on the RET) – although future experiments will have to fully test this notion. Only one previous study has examined how individuals evaluate risky effort costs. In contrast to our findings, Nagengast *et al.* reported that when there was variance associated with the effort costs, overall people were risk-seeking, rather than risk-averse^28^. Crucially, in their experiments the effort costs were *physical* rather and cognitive as in our study. This would suggest that cognitive effort might be underpinned by distinct mechanisms from physical effort. Recent research in rats supports this notion. Hosking and colleagues reported that whilst dopamine influences physical effort valuation, it does not modulate cognitive effort valuation^15^. In addition, lesions to the medial prefrontal cortex of the rat impair physical but not cognitive effort valuation^16^. Similarly, neuroimaging evidence in humans has shown distinct anatomical substrates for cognitive and physical effort processing^49^. Thus, tentatively, we suggest that our results point to a similar dissociation between cognitive and physical effort when the effort is associated with a risk, which would have important implications for the underlying neurobiology of effort processing.

Recently it has been suggested that risk-sensitivity and effort-sensitivity share common neuroanatomical and neuromodulatory mechanisms^30^. Specifically, it has been proposed that dopaminergic input from the ventral tegmental area (VTA), to the anterior cingulate cortex (ACC) and the nucleus accumbens (NAcc) is involved in guiding both risky decisions^50^,^51^,^52^ and effort-based decision-making^3^,^5^,^10^,^53^,^54^,^55^,^56^,^57^,^58^,^59^. Research in both animals and humans suggests that pharmacological interventions that modulate the amount of dopamine within this circuit modulate effort-based decision-making, and other studies have shown that dopamine antagonists and agonists modulate risky behaviors. Specifically, up-regulating dopamine function appears to increase the amount of effort that rats or humans^3^,^10^ will expend for rewards and also increase their willingness to accept risky gambles^25^. It has thus been suggested that increasing levels of dopamine in this neural circuit would increase risky behaviors and also increase motivation to overcome effort.

In contrast to this view, much research has suggested that motivation and risk-taking behaviours may be largely independent^8^,^60^. This suggests that whilst risky-decision making may share some common neural mechanisms, they are largely distinct processes. As a result risk-sensitivity and effort-discounting may be behaviorally orthogonal or dissociable, as we found here. Future research might profitably examine the relationships between different forms of cost-benefit decision-making in order to understand what mechanisms underpin disorders of motivation, such as apathy and impulsivity, and also neurological disorders which impact upon motivation^7^,^9^,^11^,^12^,^13^,^60^.

In this study we show that shifts of attention are perceived as effortful and devalue rewards, and that overall people are averse to risks in whether a high or low number of shifts of attention will have to be made. However, there was little evidence of a relationship between cognitive effort risk-aversion and effort discounting, in contrast to recent accounts of effort and risky decision-making. We suggest that our data are consistent with emerging research showing the cognitive effort processing has a distinct neurobiological signature from physical effort. Similarly effort-discounting and risky decision-making might also be underpinned by distinct neural mechanisms. Understanding motivated behavior will therefore rely on dissecting out the distinct and overlapping mechanisms that underpin different forms of cost-benefit decision-making.

## Methods

### Participants

59 healthy participants (main experiment –44, eye tracking –15) were recruited from the local area (28 male; mean = 26 years, S.D = 6.9). The study was approved by Oxford University Medical Sciences Inter-Divisional Research Ethic Committee in accordance with local guidelines and all participants gave written, informed consent. They were told that the money their payment for the experiment would vary between £8 and £12 depending on their decisions.

### Procedure

All participants initially performed a short practice session, before extensive training on the RSVP task. Following this, they performed two cost-benefit decision-making tasks in a counterbalanced order across participants.

#### Practice

During a practice session there were 24 trials of the RSVP task. They received feedback at the end of each trial informing them of the number of missed targets (failure to respond to a target within 1000 ms) and false alarm button presses (additional button presses when no target was presented). The practice began with 4 trials of only 1 shift, the number of shifts per trial then incrementally increased every 4 trials.

#### Training

In order to examine performance on the task at each level of effort and to train participants as to how effortful making shifts of attention were, 60 trials (10 trials of each effort level) of the RSVP streams were then completed in a random order. During this session participants learnt to associated a stimulus (white bar with a yellow line on it) with the amount of effort required to perform the shifts of attention and earn rewards (See supplementary methods).

In order that choices in the two decision-making tasks were driven by the perceived effort of each effort level, and not by the probability of receiving a reward, we used an adjusted reward schedule during the training session. Participants were rewarded with a credit if the combined number of misses and false alarms on a trial was 3 or fewer. However, the receipt of a credit was also fixed such that 20% of trials at each effort level were not rewarded regardless of performance, unless they made no misses and no false alarms. This approach ensured that rewards were delivered almost equally across the effort levels during the training period and that participants were under the belief that they needed to perform well enough to receive a rewarding outcome. Thus, choices during the decision-making tasks were likely to be made based on the perceived effort and not the probability of being rewarded at each effort level.

#### Effort Discounting Task (EDT)

In the Effort Discounting Task (EDT) participants were required to perform cost-benefit decisions (see Fig. 1b) between a “baseline” low effort (1 shift), low reward (1 credit) option and an “offer” that was higher in effort (2–6 shifts) and reward (2,4,6,8 or 10 credits). Across trials the reward and effort level in the offer was varied, but the baseline remained constant. 75 trials were presented in total, with 3 repetitions of each effort-reward combination. The offer and baseline were presented on the screen in the form of white bars with yellow horizontal lines on showing the effort levels and the number of credits written numerically above. Choices were made on the keyboard with one key corresponding to the left hand of the screen and the other to the right. During this task, the chosen effort was not performed and the rewards were not received. Rather, participants were informed that 10 of their trials would be selected at the end of the experiment at random and they would have to perform those RSVP trials in order to get the chosen reward. Thus, payment and the number of shifts of attention that would have to be performed would depend on their choices during the task. By performing only a selection of their choices “offline” we ensured that decisions weren’t influenced by the effects of fatigue that might accumulate during the course of an experiment where the chosen effort was performed on every trial. The EDT enabled us to index effort and reward sensitivity.

#### Risky Effort Task

In the Risky Effort Task (RET) participants were required to perform cost-benefit decisions between a “safe” option where the effort level was fixed at 3 shifts and the reward at 4 credits and a “risky” option that varied in the effort and reward levels on offer. The risky option was associated with two levels of effort, that were equally likely to be the amount of effort that would have to be performed if chosen. Across trials the risky option varied in the reward on offer (2, 4 or 6 credits) and also in terms of the variance, which could either be low (2 and 4 shifts), or high (1 and 5 shifts). There were 84 trials in total with 14 repetitions of each condition. As with the EDT task the chosen effort was not performed, but participants were instructed that 10 trials would be selected at the end of the experiment and the effort level they would have to perform and the reward on offer would depend on their choices.

This design is similar to other studies that have looked at risky decision-making in the domain of reward probability, where the average values of a safe and risky gamble are equal. Here the average amount of effort of the safe and the risky option was always 3 shifts. However, the risky option differs in terms of the variance associated with selecting the option but having to perform the higher effort option. Thus, choices of the safe option indicated risk-aversion, whereas choices of the risky option indicated risk-sensitivity.

#### Self-Reported effort

*The* NASA-TLX was completed after the decision-making tasks and tested people’s perceptions of how effortful the shifts of attention were (See supplementary methods).

#### Questionnaires

Participants completed the Urgency, Premeditation, Perseverance, Sensation Seeking, and Positive Urgency (UPPS-P) scale^29^ to examine risk-sensitivity and the LARS-e to examine apathy^2^, (See supplementary methods).

### Eye tracking control study

14 participants performed the practice and training sessions of the main experiment whilst undergoing eye-tracking (see supplementary methods).

## Additional Information

**How to cite this article**: Apps, M. A. J. *et al.* The role of cognitive effort in subjective reward devaluation and risky decision-making. *Sci. Rep.* **5**, 16880; doi: 10.1038/srep16880 (2015).

## Supplementary Material

Supplementary Information

## References

[b1] Hull C. Principles of behavior. (Appleton-Century, 1943).

[b2] Bonnelle V. *et al.* Characterization of reward and effort mechanisms in apathy. J. Physiol.-Paris 109, 16–26 (2014). 24747776 10.1016/j.jphysparis.2014.04.002 PMC4451957

[b3] Phillips P. E. M., Walton M. E. & Jhou T. C. Calculating utility: preclinical evidence for cost-benefit analysis by mesolimbic dopamine. Psychopharmacology (Berl.) 191, 483–495 (2007). 17119929 10.1007/s00213-006-0626-6

[b4] Charnov E. L. Optimal foraging, marginal value theorem. Theor. Popul. Biol. 9, 129–136 (1976). 1273796 10.1016/0040-5809(76)90040-x

[b5] Salamone J. D., Correa M., Farrar A. & Mingote S. M. Effort-related functions of nucleus accumbens dopamine and associated forebrain circuits. Psychopharmacology (Berl.) 191, 461–482 (2007). 17225164 10.1007/s00213-006-0668-9

[b6] Niv Y. Cost, benefit, tonic, phasic: what do response rates tell us about dopamine and motivation? Ann. N. Y. Acad. Sci. 1104, 357–376 (2007). 17416928 10.1196/annals.1390.018

[b7] Adam R. *et al.* Dopamine reverses reward insensitivity in apathy following globus pallidus lesions. Cortex 49, 1292–1303 (2013). 22721958 10.1016/j.cortex.2012.04.013 PMC3639369

[b8] Sinha N., Manohar S. & Husain M. Impulsivity and apathy in Parkinson’s disease. J. Neuropsychol. 7, 255–283 (2013). 23621377 10.1111/jnp.12013 PMC3836240

[b9] Bari A. & Robbins T. W. Inhibition and impulsivity: behavioral and neural basis of response control. Prog. Neurobiol. 108, 44–79 (2013). 23856628 10.1016/j.pneurobio.2013.06.005

[b10] Chong T. T.-J. *et al.* Dopamine enhances willingness to exert effort for reward in Parkinson’s disease. Cortex. 69, 40–46 (2015). 25967086 10.1016/j.cortex.2015.04.003 PMC4533227

[b11] Hartmann M. N. *et al.* Apathy but not diminished expression in schizophrenia is associated with discounting of monetary rewards by physical effort. Schizophr. Bull. 41, 503–512 (2015). 25053653 10.1093/schbul/sbu102 PMC4332944

[b12] Van Reekum R., Stuss D. T. & Ostrander L. Apathy: Why care? J. Neuropsychiatry Clin. Neurosci. 17, 7–19 (2005). 15746478 10.1176/jnp.17.1.7

[b13] Robert P. H. b, Mulin E., Malléa P. & David R. Apathy diagnosis, assessment, and treatment in Alzheimer’s disease. CNS Neurosci. Ther. 16, 263–271 (2010). 20345973 10.1111/j.1755-5949.2009.00132.x PMC6493869

[b14] Clarke D. E., Ko J. Y., Lyketsos C., Rebok G. W. & Eaton W. W. Apathy and cognitive and functional decline in community-dwelling older adults: results from the Baltimore ECA longitudinal study. Int. Psychogeriatr. 22, 819–829 (2010). 20478091 10.1017/S1041610209991402 PMC2893259

[b15] Hosking J. G., Floresco S. B. & Winstanley C. A. Dopamine Antagonism Decreases Willingness to Expend Physical, but not Cognitive, Effort: a Comparison of Two Rodent Cost/Benefit Decision-Making Tasks. Neuropsychopharmacology. (2014). 10.1038/npp.2014.285 PMC4330516 25328051

[b16] Hosking J. G., Cocker P. J. & Winstanley C. A. Prefrontal Cortical Inactivations Decrease Willingness to Expend Cognitive Effort on a Rodent Cost/Benefit Decision-Making Task. Cereb. Cortex (2015). 10.1093/cercor/bhu321 PMC4785948 25596594

[b17] Kool W., McGuire J. T., Rosen Z. B. & Botvinick M. M. Decision Making and the Avoidance of Cognitive Demand. J. Exp. Psychol. Gen. 139, 665–682 (2010). 20853993 10.1037/a0020198 PMC2970648

[b18] Westbrook A., Kester D. & Braver T. S. What is the subjective cost of cognitive effort? Load, trait, and aging effects revealed by economic preference. PloS One 8, (2013). 10.1371/journal.pone.0068210 PMC3718823 23894295

[b19] Kool W. & Botvinick M. A labor/leisure tradeoff in cognitive control. J. Exp. Psychol. Gen. 143, 131–141 (2014). 23230991 10.1037/a0031048 PMC3739999

[b20] Birnbaum M. H. New paradoxes of risky decision making. Psychol. Rev. 115, (2008). 10.1037/0033-295X.115.2.463 18426300

[b21] Quartz S. R. Reason, emotion and decision-making: risk and reward computation with feeling. Trends Cogn. Sci. 13, 209–215 (2009). 19362037 10.1016/j.tics.2009.02.003

[b22] Kahneman D. & Tversky A. Choices, values, and frames. Am. Psychol. 39, 341–350 (1984).

[b23] Tversky A. & Kahneman D. Advances in prospect theory: Cumulative representation of uncertainty. J. Risk Uncertain. 5, 297–323 (1992).

[b24] Stewart N., Chater N., Stott H. P. & Reimers S. Prospect relativity: how choice options influence decision under risk. J. Exp. Psychol. Gen. 132, (2003). 10.1037/0096-3445.132.1.23 12656296

[b25] Norbury A., Manohar S., Rogers R. D. & Husain M. Dopamine modulates risk-taking as a function of baseline sensation-seeking trait. J. Neurosci. 33, 12982–12986 (2013). 23926253 10.1523/JNEUROSCI.5587-12.2013 PMC3735881

[b26] Rogers R. D. *et al.* Dissociable deficits in the decision-making cognition of chronic amphetamine abusers, opiate abusers, patients with focal damage to prefrontal cortex, and tryptophan-depleted normal volunteersEvidence for monoaminergic mechanisms. Neuropsychopharmacology 20, 322–339 (1999). 10088133 10.1016/S0893-133X(98)00091-8

[b27] Pyke G. H. Optimal foraging theory: a critical review. Annual Review of Ecology and Systematics, 15, 523–575 (1984).

[b28] Nagengast A. J., Braun D. A. & Wolpert D. M. Risk-sensitivity and the mean-variance trade-off: Decision making in sensorimotor control. Proc. R. Soc. B Biol. Sci. 278, 2325–2332 (2011). 10.1098/rspb.2010.2518 PMC3119020 21208966

[b29] Whiteside S. P., Lynam D. R., Miller J. D. & Reynolds S. K. Validation of the UPPS impulsive behaviour scale: a four-factor model of impulsivity. Eur. J. Personal. 19, 559–574 (2005).

[b30] Miller M. A., Thome A. & Cowen S. L. Intersection of effort and risk: ethological and neurobiological perspectives. Front. Neurosci. 7 (2013). 10.3389/fnins.2013.00208 PMC3819579 24223535

[b31] Yantis S. *et al.* Transient neural activity in human parietal cortex during spatial attention shifts. Nat. Neurosci. 5, 995–1002 (2002). 12219097 10.1038/nn921

[b32] Hart S. G. & Staveland L. E. in Human mental workload (North Holland Press, 1988).

[b33] Hartmann M. N. *et al.* Apathy in Schizophrenia as a Deficit in the Generation of Options for Action. J. Abnorm. Psychol. (2015). 10.1037/abn0000048 25688424

[b34] Sockeel P. B. *et al.* The Lille apathy rating scale (LARS), a new instrument for detecting and quantifying apathy: Validation in Parkinson’s disease. J. Neurol. Neurosurg. Psychiatry 77, 579–584 (2006). 16614016 10.1136/jnnp.2005.075929 PMC2117430

[b35] Kurzban R., Duckworth A., Kable J. W. & Myers J. An opportunity cost model of subjective effort and task performance. Behav. Brain Sci. 36, 661–679 (2013). 24304775 10.1017/S0140525X12003196 PMC3856320

[b36] Westbrook A. & Braver T. S. Cognitive effort: A neuroeconomic approach. Cogn. Affect. Behav. Neurosci. 15, 395–341 (2015). 25673005 10.3758/s13415-015-0334-y PMC4445645

[b37] Klein-Flugge M. C., Kennerley S. W., Saraiva A. C., Penny W. D. & Bestmann S. Behavioral modeling of human choices reveals dissociable effects of physical effort and temporal delay on reward devaluation. PLoS Comput. Biol. 11, (2015). 10.1371/journal.pcbi.1004116 PMC4376637 25816114

[b38] Mazur J. E. Choice between single and multiple delayed reinforcers. J. Exp. Anal. Behav. 46, 67–77 (1986). 3746189 10.1901/jeab.1986.46-67 PMC1348257

[b39] Mazur J. E. Hyperbolic value addition and general models of animal choice. Psychol. Rev. 108, 96–112 (2001). 11212635 10.1037/0033-295x.108.1.96

[b40] Prevost C., Pessiglione M., Metereau E., Clery-Melin M.-L. & Dreher J.-C. Separate Valuation Subsystems for Delay and Effort Decision Costs. J. Neurosci. 30, 14080–14090 (2010). 20962229 10.1523/JNEUROSCI.2752-10.2010 PMC6634773

[b41] Hajcak G. & Foti D. Errors are aversive: defensive motivation and the error-related negativity. Psychol. Sci. 19, 103–108 (2008). 18271855 10.1111/j.1467-9280.2008.02053.x

[b42] Shadmehr R., De Xivry J. J. O., Xu-Wilson M. & Shih T. Y. Temporal Discounting of Reward and the Cost of Time in Motor Control. J. Neurosci. 30, 10507–10516 (2010). 20685993 10.1523/JNEUROSCI.1343-10.2010 PMC2926660

[b43] Shadmehr R. Control of movements and temporal discounting of reward. Curr. Opin. Neurobiol. 20, 726–730 (2010). 20833031 10.1016/j.conb.2010.08.017

[b44] Manohar S. G. *et al.* Reward Pays the Cost of Noise Reduction in Motor and Cognitive Control. Curr. Biol. 25, 1707–1716 (2015). 26096975 10.1016/j.cub.2015.05.038 PMC4557747

[b45] DeSimone J. C., Everling S. & Heath M. The antisaccade task: visual distractors elicit a location-independent planning ‘cost’. PloS One 10, (2015). 10.1371/journal.pone.0122345 PMC4382315 25830383

[b46] Weiler J., Hassall C. D., Krigolson O. E. & Heath M. The unidirectional prosaccade switch-cost: Electroencephalographic evidence of task-set inertia in oculomotor control. Behav. Brain Res. 278, 323–329 (2015). 25453741 10.1016/j.bbr.2014.10.012

[b47] Chiau H.-Y. *et al.* Trial type probability modulates the cost of antisaccades. J. Neurophysiol. 106, 515–526 (2011). 21543748 10.1152/jn.00399.2010 PMC3154828

[b48] Chiu Y.-C. & Yantis S. A domain-independent source of cognitive control for task sets: shifting spatial attention and switching categorization rules. J. Neurosci. 29, 3930–3938 (2009). 19321789 10.1523/JNEUROSCI.5737-08.2009 PMC2817948

[b49] Schmidt L., Lebreton M., Clery-Melin M.-L., Daunizeau J. & Pessiglione M. Neural Mechanisms Underlying Motivation of Mental Versus Physical Effort. Plos Biol. 10, (2012). 10.1371/journal.pbio.1001266 PMC3283550 22363208

[b50] Norbury A. & Husain M. Sensation-seeking: Dopaminergic modulation and risk for psychopathology. Behav. Brain Res. 288, 79–93 (2015). 25907745 10.1016/j.bbr.2015.04.015

[b51] Floresco S. B., Onge J. R. S., Ghods-Sharifi S. & Winstanley C. A. Cortico-limbic-striatal circuits subserving different forms of cost-benefit decision making. Cogn. Affect. Behav. Neurosci. 8, 375–389 (2008). 19033236 10.3758/CABN.8.4.375

[b52] Rushworth M. F. S. & Behrens T. E. J. Choice, uncertainty and value in prefrontal and cingulate cortex. Nat. Neurosci. 11, 389–397 (2008). 18368045 10.1038/nn2066

[b53] Gan J. O., Walton M. E. & Phillips P. E. M. Dissociable cost and benefit encoding of future rewards by mesolimbic dopamine. Nat. Neurosci. 13, 25–27 (2010). 19904261 10.1038/nn.2460 PMC2800310

[b54] Apps M. A. & Ramnani N. The Anterior Cingulate Gyrus Signals the Net Value of Others’ Rewards. J. Neurosci. 34, 6190–6200 (2014). 24790190 10.1523/JNEUROSCI.2701-13.2014 PMC4004808

[b55] Pessiglione M. *et al.* How the brain translates money into force: a neuroimaging study of subliminal motivation. Science 316, 904–906 (2007). 17431137 10.1126/science.1140459 PMC2631941

[b56] Schouppe N., Demanet J., Boehler C. N., Ridderinkhof K. R. & Notebaert W. The role of the striatum in effort-based decision-making in the absence of reward. J. Neurosci. 34, 2148–2154 (2014). 24501355 10.1523/JNEUROSCI.1214-13.2014 PMC6608538

[b57] Vassena E. *et al.* Overlapping neural systems represent cognitive effort and reward anticipation. PloS One 9, (2014). 10.1371/journal.pone.0091008 PMC3946624 24608867

[b58] Kurniawan I. T. *et al.* Choosing to Make an Effort: The Role of Striatum in Signaling Physical Effort of a Chosen Action. J. Neurophysiol. 104, 313–321 (2010). 20463204 10.1152/jn.00027.2010 PMC2904211

[b59] Burke C. J., Brunger C., Kahnt T., Park S. Q. & Tobler P. N. Neural Integration of Risk and Effort Costs by the Frontal Pole: Only upon Request. J. Neurosci. 33, 1706–+ (2013). 23345243 10.1523/JNEUROSCI.3662-12.2013 PMC6618754

[b60] Dalley J. W., Everitt B. J. & Robbins T. W. Impulsivity, Compulsivity, and Top-Down Cognitive Control. Neuron 69, 680–694 (2011). 21338879 10.1016/j.neuron.2011.01.020

[b61] Hartmann M. N., Hager O. M., Tobler P. N. & Kaiser S. Parabolic discounting of monetary rewards by physical effort. Behav. Processes 100, 192–196 (2013). 24140077 10.1016/j.beproc.2013.09.014

