# Supplementary material for: The role of cognitive effort in subjective reward devaluation and risky decision-making
Source: Sci Rep. 2015 Nov 20;5:16880. doi: 10.1038/srep16880 (PMC4653618; doi:10.1038/srep16880)
Supplement: Supplementary Information [file srep16880-s1.pdf]

# The role of cognitive effort in subjective reward devaluation and risky decision-making

Matthew A J Apps[1,2], Laura Grima[2], Sanjay Manohar[2], Masud Husain[1,2]

[1]Nuffield Department of Clinical Neuroscience, University of Oxford, Oxford, UK

[2] Department of Experimental Psychology, University of Oxford, Oxford, UK

## Supplementary Methods

### *Participants*

Four participants were excluded from the main experiment and one from the eye tracking study due to poor task performance (<40% credits received during training), medication, or current psychological treatment.

### *Apparatus*

All experiments were conducted on a PC running on Presentation software (Neurobehavioral Systems) which was used for stimulus presentation and response collection. Self-report questionnaires were completed by hand. Analyses were performed using Matlab 2013b (Mathworks inc.) and SPSS 20 (IBM). For the additional eye-tracking experiment, an EyeLink 1000 Hz desk-mounted monocular infra-red eye-tracker was used to record eye movements.

### Stimulus presentation

The peripheral streams were offset at a visual angle of 2° from the central array, based on participants sitting at a distance of approximately 80 cm from the screen. Three distractor streams flanked each of the peripheral streams – one above, one below, and one to the side of the streams. These distractor streams displayed task-irrelevant stimuli to increase the difficulty of the task. The stimuli consisted of an assortment of white randomly selected letters, presented in Times New Roman font size 18, and measuring approximately 0.38° against a black background. In pilot studies, we determined the positioning of the target streams and the central stream to ensure that only the central and peripheral stream could be attended simultaneously, with the target stream on the other side of central fixation positioned in a position that could not be attended with both the central stream and the contralateral target stream. This ensured that they would have to make shifts of attention to detect targets.

### Training Session

Each trial began with a white bar with a yellow line positioned horizontally across the bar. The height of the yellow line on the bar indicated how many shifts there would be in the upcoming trial. Participants were therefore able to learn the associations between the amount of effort that they need to exert and the height of the yellow line. Trials were

presented in a pseudorandom order, with no more than 2 repetitions of each effort level in the sequence of trials. In contrast to the initial practice session, no explicit feedback about the number of false alarms and misses was presented. Instead at the end of each trial they were provided with feedback in the form of one or zero credits depending upon performance. Participants were instructed that they would receive a credit if they had performed “well enough” at the task. All RSVP trials were fixed at the same duration of 14s, regardless of the duration to avoid any effects of temporal discounting. This approach did introduce additional potential sources of effort – such as longer periods of sustained attention in the lower effort level. However, the self-report ratings from the NASA-TLX suggests that any such changes in sustained attention were not influencing the perception of effort (see fig.2).

The task was designed to remain as consistent as with the work of Yantis et al., (2002) as possible. During the task numerals were used as targets and switch cues (“3” or “7”). These numbers were arbitrarily chosen and conveyed no specific meaning to the participants. The same cues were used for all subjects.

Importantly, to ensure that at all effort levels participants were maintaining a central fixation and attending to a peripheral stream to targets, all effort levels required switches of attention. That is, for the lowest effort level we did not use 0 switches, as such a condition would not require participants to maintain a central fixation and attend to two streams simultaneously. Thus, such a condition would not be comparable to the other effort levels. For this reason we used 1 switch as the lowest effort level.

To ensure that participants were not able to employ any strategy to detect targets, the occurrence of targets and switch cues were fully randomised on each trial, such that each participant would not be able to predict the occurrence of a target or switch. The only constraints that were applied to the order of stimulus presentation were as follows. Target (“7”) or shift (“3”) stimuli could not re-occur within 700ms, nor could they be within 700ms of each other. This ensured that there was sufficient time to respond to the target stimuli and switch their attention before another stimulus requiring a response was presented. In addition, the shifts could not be in the first or final 1050ms of a trial and the targets could not be in the first 1050ms of the trial. All stimuli presented in the array were changed

randomly to a different letter or numeral every 350ms. However, all other aspects of the presentation of letters, targets and shifts were randomised.

**NASA-TLX**

To verify that the RSVP trials were experienced as effortful and that this increased as the number of shifts in the RSVP streams increased, participants were asked to provide ratings for each effort level on the questions of the NASA-Task Load Index (NASA-TLX). In the NASA-TLX the following questions were asked in reference to each effort level:

1. How mentally demanding was this? (mental)
2. How physically demanding was this? (physical)
3. How hurried or rushed was this? (temporal)
4. How successful were you in accomplishing this? (performance)
5. How hard did you have to work to accomplish your performance at this level? (effort)
6. How insecure, discouraged, irritated, stressed and annoyed were you at this? (frustration)

Responses were given on a scale between -10 (very low) and +10 (very high) for each question for each level of effort. This questionnaire provided a useful tool for understanding how effortful and specifically mentally demanding participants found the task.

*Questionnaires*

The UPPS-P is a 59-point questionnaire that measures trait levels of impulsivity and risk-taking on a Likert scale which ranges from 1 ('agree strongly') to 4 ('disagree strongly'). The LARS-e (Lille Apathy Ratings Scale extended) is an apathy questionnaire, version of the LARS clinical interview, adapted for self-completion in the healthy population. The LARS-e is a 50 item questionnaire answered on a 1-5 scale from 'strongly disagree' to 'strongly agree'.

**Eye-Tracking control study**

Stimuli were displayed via Presentation on a CRT at 1024 × 768 pixels. Participants performed 9-point calibration at the start of the experiment. Participants had to fixate a central cross before the start of each trial. Monocular eye-movement recordings were obtained at 1000 Hz for the duration of the experiment. Participants performed the same practice and training trials as had been performed during the main experiment. Saccades

were extracted automatically using criteria of velocity > 30 $°s^{-1}$, acceleration > 8000 $°s^{-2}$, and amplitude > 1.5° of visual angle, for the 14 s duration of each trial. The number saccades in each trial were calculated to exclude motor effort costs as a driving factor. Locations of the intervening fixations were binned into a two-dimensional spatial histogram for each participant. The histogram was convolved with a 0.3 degree Gaussian kernel to form a heatmap of fixations (Fig 4).

**Supplementary Results**

*Task performance*

To examine whether participants were able to meet the criteria and successfully obtain rewards, at the different effort levels, an ANOVA on the proportion of rewards obtain at each each effort level was performed. This revealed a significant effect of effort level on the proportion of rewards obtained ($F(5, 120) = 11.1$, $p < 0.001$). However, we found that this effect was better explained by a cubic trend ($F(1,38) = 13.33$, $p < 0.001$), than a linear trend ($F(1,38) = 4.42$, $p < 0.05$), suggesting that the probability of receiving a reward did not linearly increase with the effort level. Moreover, overall participants were on average successful at meeting the criteria required

A repeated measures ANOVA revealed a significant decline in d’ scores (hits-false alarms) as the effort level increased. Although overall participants were very successful, with the average d’ scores greater than 2.1, even at the highest effort level (Supp. Fig.1a). Moreover, the average percentage of trials on which participants successfully reached the criteria to obtain a reward was greater than 93% even at the highest effort level.

The number of button presses were also significantly different across the effort levels ($F(4.0, 157.3) = 3.11$, $p < 0.017$) and there was also a marginally significant negative correlation between the mean number of button presses and success ($R_s = -0.75$, $p = 0.08$). However, we found that the number of button presses decreased as the effort level increased, perhaps explaining the positive relationship between button presses and choice on the EDT.

To address whether there was a time-on-task effect we correlated trial number with the number button presses made on each trial and also the success. We found no correlation between button presses or success and trial number (Button presses: $R_s = -0.026$, $p = 0.84$; Success: $R_s = -0.093$, $p = 0.48$). Thus, it seems there was no time-on-task effect on the

performance of the effort task. We have now included this information in supplementary results.

Choice behavior.

To examine whether there was an order effect on the two tasks we compared participants choice behavior who performed the EDT first with those who performed it second. We found no difference in reward or effort t-scores (Effort: $t(36.5) = -1$, $p > 0.34$; Reward: $t(37.78) = -0.86$, $p > 0.39$). Similarly, we found no difference in risk or reward t-scores from those who performed the RET first or second (Reward: $t(37.99) = -1.02$, $p > 0.3$; Risk: $t(29.38) = 0.33$, $p > 0.75$).

It is important to note that we did not examine ANOVAs containing both effort and reward for the EDT. This was due to the fact that there were only 3 repetitions of each cell within the 5x5 design. Such a design makes examining Effort X Reward interactions inappropriate.

To avoid issues with collinearity within the logistic regressions used to analyse the EDT and RET choice behavior we did not include any interaction terms. We note however, that performing these supplementary analyses did not reveal a significant effect of a Risk X Reward interaction on the RET or the Effort X Reward interaction on the EDT ($p > 0.05$).

## Supplementary Figure

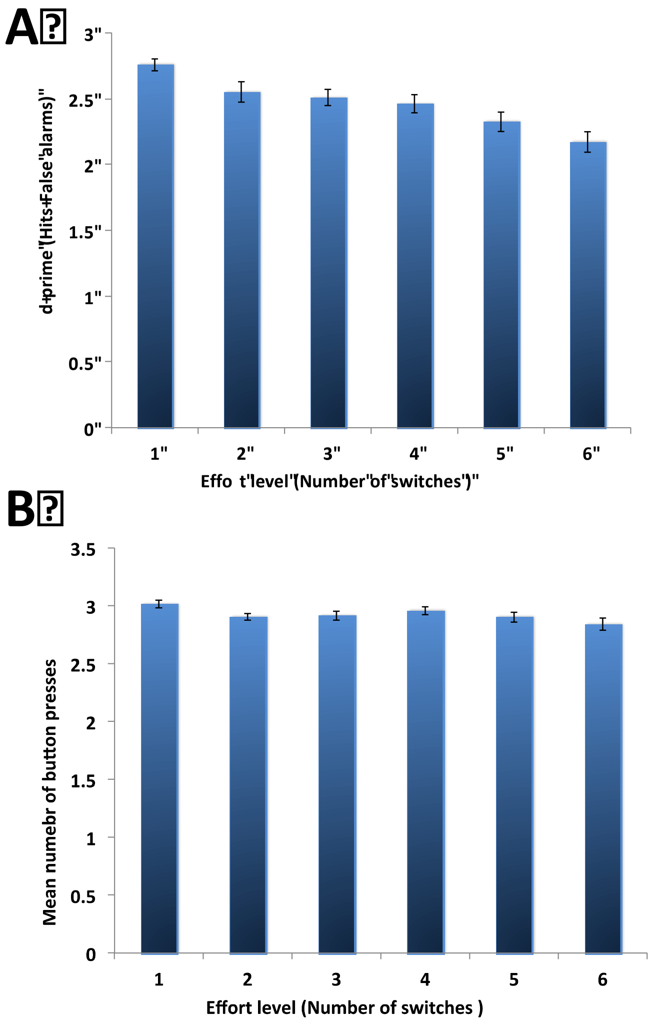


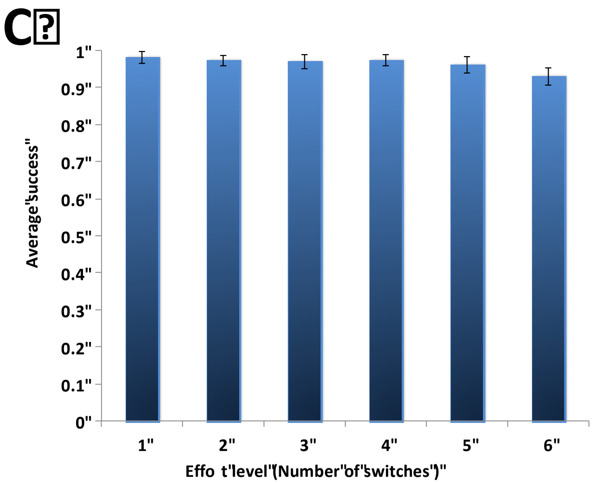


Supp fig.1 Performance on the RSVP task. (A) Average D' scores declined as the number of switches increased. (B) Mean number of button presses decreased across effo t levels. (C) Success (mean number of false alarms + misses).
